# Current News

**Published:** 1904-02

**Authors:** 


### Current News.

### THE NEW YORK INSTITUTE OF STOMATOLOGY.

THE following officers of The New York Institute of Stomatology have been elected for 1904:

President, A. H. Brockway; Vice-President, C. O. Kimball; Treasurer, J. Adams Bishop; Recording Secretary, H. L. Wheeler; Corresponding Secretary, J. B. Locherty; Curator, S. H. McNaughton; Editor, F. L. Bogue; Past President, J. Morgan Howe.

*Executive Committee.*—S. E. Davenport, Chairman; Charles F. Allan, F. Milton Smith.

### SOUTHERN BRANCH, NATIONAL DENTAL ASSOCIATION.

THE next meeting of the Southern Branch of the National Dental Association will be held in Washington, D. C., February 23 to 26, inclusive. The Association will meet conjointly with the District of Columbia and Maryland State Dental Associations, in response to an invitation from those local organizations.

The opening meeting will be held in Columbian University Hall, Fifteenth, corner H Street, N. W. The clinics and other meetings of the Association will be held in the Medical and Dental Department building of Columbian University on H Street, between Thirteenth and Fourteenth Streets. There will be a banquet at the New Willard Hotel on Thursday evening, February 25, given by the Maryland State Dental Association and the District of Columbia Dental Society, complimentary to the Southern Branch of the National Dental Association as their guests.

The Southeastern Passenger Association grants a rate of one and one-third fare, plus twenty-five cents, from all points in territory south of the Ohio and Potomac and east of the Mississippi. The hotels of Washington have granted reduced rates, as follows:

The New Willard (head-quarters), $2.50 and up, European plan; the Raleigh, $2 and up, European plan; the Ebbitt, $3 and up, American plan; the Riggs, $3 and up, American plan; the Oxford, $2 and up, American plan; the Hamilton, $2 and up, American plan.

Ample provision will be made at the University for clinics and exhibits. All practitioners who conduct themselves according to the code of ethics are cordially invited to attend.

L. G. NOEL,
*Chairman Programme Committee.*

[The foregoing notice of the Southern Branch of the National Dental Association was received as we were about to go to press, and too late for the insertion of the list of papers and clinics. This is an unusually full one, indicating the promise of an exceedingly interesting meeting at Washington, D. C.—ED.]

